# Lifelong Learning and Community Empowerment Among Older Chinese Immigrants in Canada

**DOI:** 10.1093/geroni/igaf122.864

**Published:** 2025-12-31

**Authors:** Weiguo Zhang

**Affiliations:** University of Toronto, Toronto, Ontario, Canada

## Abstract

Lifelong learning is essential for encouraging social inclusion, resilience, and empowerment among older adults, especially in immigrant communities. The Chinese Age-well Research and Education (CARE) initiative tackles key challenges faced by older Chinese immigrants in Canada through several projects. The Intergenerational Dialogue on Elder Abuse project builds awareness, provides emotional support, and strengthens ties between generations to prevent older adult’s mistreatment. The Intergenerational and Intercultural Dialogue to Fight Racism project teaches older Chinese immigrants about systemic racism, how it connects with ageism, and ways to respond to discrimination. CARE also supports digital literacy and storytelling, giving older adults tools to share their stories and connect with wider social issues. These efforts show how working across generations and offering focused education help older Chinese immigrants address social challenges, create strong support networks, and take part in community advocacy. Many older immigrants deal with language difficulties, cultural gaps, and struggles to recognize or handle discrimination, making accessible and culturally sensitive learning vital. Through research, education, and community collaboration, CARE uses lifelong learning as a means of social change, emphasizing the need for inclusive policies and programs to support the well-being and active involvement of older immigrant.

